# RFI Artefacts Detection in Sentinel-1 Level-1 SLC Data Based On Image Processing Techniques

**DOI:** 10.3390/s20102919

**Published:** 2020-05-21

**Authors:** Agnieszka Chojka, Piotr Artiemjew, Jacek Rapiński

**Affiliations:** 1 Faculty of Geoengineering, University of Warmia and Mazury in Olsztyn, 10-719 Olsztyn, Poland; jacek.rapinski@uwm.edu.pl; 2 Faculty of Mathematics and Computer Science, University of Warmia and Mazury in Olsztyn, 10-719 Olsztyn, Poland; artem@matman.uwm.edu.pl

**Keywords:** RFI, artefacts, InSAR, image processing, pixel convolution, thresholding, nearest neighbor filtering, deep learning

## Abstract

Interferometric Synthetic Aperture Radar (InSAR) data are often contaminated by Radio-Frequency Interference (RFI) artefacts that make processing them more challenging. Therefore, easy to implement techniques for artefacts recognition have the potential to support the automatic Permanent Scatterers InSAR (PSInSAR) processing workflow during which faulty input data can lead to misinterpretation of the final outcomes. To address this issue, an efficient methodology was developed to mark images with RFI artefacts and as a consequence remove them from the stack of Synthetic Aperture Radar (SAR) images required in the PSInSAR processing workflow to calculate the ground displacements. Techniques presented in this paper for the purpose of RFI detection are based on image processing methods with the use of feature extraction involving pixel convolution, thresholding and nearest neighbor structure filtering. As the reference classifier, a convolutional neural network was used.

## 1. Introduction

Since NASA launched the first satellite (at that time known as the Earth Resources Technology Satellite) of the United States’ Landsat program in 1972 [1], satellite remote sensing has developed strongly. They are currently placing newer satellites in orbit around Earth, equipped with advanced sensors for Earth monitoring. Among them worthy of emphasis are satellites with Synthetic Aperture Radar (SAR) instruments on board.

SAR is an active microwave remote sensing tool [2] with wide spatial coverage, fine resolution, all-weather and day-and-night image acquisition capability [3,4]. These features allow the ability to use SAR images for a multitude of scientific research, commercial and defense applications ranging from geoscience to Earth system monitoring [2,4,5].

Interferometric Synthetic Aperture Radar (InSAR) technology exploits differences in the phase of the waves returning to the satellite of at least two complex SAR images to generate, for instance, surface topography and deformation maps or Digital Elevation Models (DEMs) [6,7,8].

Among various InSAR techniques, satellite Differential Interferometric Synthetic Aperture Radar (DInSAR) has emerged as a powerful instrument to measure surface deformation associated with ground subsidence on a large scale with centimeter to millimeter accuracy [9,10,11].

DInSAR exploits the phase information of SAR images to calculate the ground displacements between two different satellite acquisitions [12]. The Permanent Scatterers InSAR (PSInSAR) method, an upgrade of DInSAR, for analytical purposes uses long stacks of co-registered SAR images to identify coherent points that provide consistent and stable response to the radar on board of a satellite [13]. Phase information obtained from these persistent scatterers is used to derive the ground displacement information and its temporal evolution [9].

Thanks to the increasing availability of a large amount of SAR data from such missions like ALOS-2, COSMO-SkyMed, PAZ, RADARSAT-2, Sentinel-1 or TerraSAR-X [3,11] and due to high-quality images covering a wide area [14,15], in the last decades, Earth observation techniques have become a valuable and indispensable remote sensing tool in geophysical monitoring of natural hazards such as earthquakes [16], volcanic activity [17] or landslides [18], mine subsidence monitoring [19] and structural engineering, especially monitoring of subsidence [20] and structural stability of buildings [15] or bridges [10].

DInSAR and PSInSAR are complementary methods and both have essential advantages and some disadvantages [16]. By way of illustration, PSInSAR is considered to be more precise than DSInSAR, because it requires about 15–20 SAR data acquisitions for a successful result, whereas DInSAR needs only 2 [13].

Despite the obvious benefits, InSAR technology has some limitations. InSAR measurements are often affected by various artefacts that not only make interpreting them more challenging, but also affect the reliability and accuracy of its outcomes.

One of the most significant is the effect of the atmosphere. In general, it results from the phenomenon of electromagnetic waves delay when traveling through the troposphere and accelerate when traveling through the ionosphere [3]. Atmospheric artefacts are usually strongly correlated with the topography (elevation) and the proximity of the sea [11,21]. Over the past decades, numerous methods were investigated to identify and mitigate these artefacts e.g., [3,8,11,22,23].

Another type of InSAR data failure described in the literature is a border noise [24,25,26,27]. This undesired processing artefact appeared in all of the Sentinel-1 GRD products generated before March 2018 [24]. Although this problem has been solved for the newly generated products, it did not cover the entire range of products and researchers still develop new methods and tools to effectively detect and remove this particular type of noise [24,25,26,27].

Contrary to the unwanted artefacts influence on InSAR data, Bouvet [28] proposed a new indicator of deforestation based on geometric artefact, called the shadowing effect. It appears in SAR images in the form of a shadow at the border of the deforested patch.

The primary issue of this paper is one more contamination frequently appearing in SAR images that is called Radio-Frequency Interference (RFI). Since the work is in progress on the development of the automatic monitoring system for high-energy para-seismic events, the urgent matter is to elaborate the effective method supporting the automatic PSInSAR processing workflow by the removal of faulty SAR data (with artefacts) that can lead to misinterpretation of the final results.

Our main goal is to find the easiest to implement technique for marking images with RFI artefacts. The solution presented in this paper for the purpose of RFI detection was based on image processing methods with the use of feature extraction involving pixel convolution, thresholding and nearest neighbor structure filtering techniques. As the reference classifier we used a convolutional neural network.

After a short introduction on the characteristics of RFI, common forms and sources of this artefact, included in Section 2, the used materials are presented in Section 3. The applied methodology and techniques are depicted in Section 4. Their results are presented and comprehensively discussed in Section 5. The main findings and recommendations for future work are summarized in Section 6.

## 2. RFI Artefact

RFI is defined as ’the effect of unwanted energy due to one or a combination of emissions, radiations or inductions upon reception in a radio communication system, manifested by any performance degradation, misinterpretation or loss of information which could be extracted in the absence of such unwanted energy’ according to Article 1.166 of the International Telecommunication Union Radio Regulations [29].

In the SAR images these incoherent electromagnetic interference signals usually appear as various kind of bright linear features [30,31] like bright stripes with curvature or dense raindrops [2,4]. RFI introduces artefacts in image by slight haziness [2,31] that acutely degrade its quality [4,30]. Such affected images may lead to wrong interpretation process and results [2].

The reason for RFI contamination for SAR is that many different radiation sources operate with the same frequency band as the SAR system [2,30]. In general they can be grouped into terrestrial and space-borne sources [2]. Most of these incoherent electromagnetic interference signals are emitted by terrestrial commercial or industrial radio devices [2], e.g., communication systems, television networks, air-traffic surveillance radars, meteorological radars, radiolocation radars, amateur radios and other, mainly military-based, radiation sources [2,4,30,31]. An example of space-borne RFI source are signals broadcasting from other satellites, such as global navigation satellite systems (GNSSs) constellations, communication satellites or other active remote sensing systems [2].

Over the past years, great efforts have been made to better understand RFI effects and to develop robust methods for detecting and mitigating this artefact, e.g., [32,33,34,35,36,37], in particular from SAR data. See [2] for general review. Although in the majority of cases, research is focused on the recognition and removal of RFI signatures from L-band SAR data, where this artefact is commonly observed [30]. In case of SAR systems, the most susceptible for RFI effects are signals operating in the low band frequency region, such as P, L, S and even C-band [2,30,31]. Studies are usually conducted on raw data [2,4,30,31,38]. To fill this gap in the literature, our research addressed the detection of RFI artefacts in SAR data, especially in recently available Sentinel-1 data. Additionally, our work meets the recommendations, proposed among others by Tao [2] and Itschner [39], concerning the application of artificial intelligent techniques, such as deep learning methods, for RFI recognition.

## 3. Materials

This section shortly describes the materials we used in our investigation. We depict the source data and datasets used to carry out experiments and tests of our solution.

### 3.1. Source Data

A set of dedicated satellites, the Sentinel families, are developed by the European Space Agency (ESA) for the operational needs of the Copernicus programme [40]. The European Union’s Earth observation programme delivers operational data and information services openly and freely in a wide range of applications in a variety of areas, such as urban area management, agriculture, tourism, civil protection, infrastructure and transport [41].

The Sentinel-1 mission is a polar-orbiting, all-weather, day-and-night C-band synthetic aperture radar imaging mission for land and ocean services. It is based on a constellation of two satellites: Sentinel-1A was launched on 3 April 2014 and Sentinel-1B on 25 April 2016 [40,42].

Sentinel-1 data are intended to be available systematically and free of charge, without limitations, to all data users including the general public, scientific and commercial users [40]; however, the most reachable for the majority of users are Level-1 products, provided as Single Look Complex (SLC) and Ground Range Detected (GRD) [40].

Level-1 Single Look Complex (SLC) products, as other Sentinel-1 data products, are delivered as a package containing, among others, metadata, measurement data sets and previews [40].

The Sentinel-1 SAR instrument mainly operates in the Interferometric Wide (IW) swath mode over land [26,40] that is one of four Sentinel-1 acquisition modes. IW uses the Terrain Observation with Progressive Scans SAR (TOPSAR) technique [43] and provides data with a large swath width of 250 km at 5 m × 20 m (range × azimuth) spatial resolution for single look data.

In this study, we used quick-look images contained in the preview folder, a part of Sentinel-1 data package. As in our case we have dual polarization products, data are represented by a single composite color image in RGB [40]. Because quick-look data has a lower resolution version of the source image, it is easy to detect RFI artefacts and then exclude data contaminated by RFI from further processing, thus improving the whole PSInSAR processing workflow, which in our case required a stack of 25–30 SAR images to calculate the ground displacements. Faulty data included in this dataset would lead to misinterpretation of the processing results. Besides, elimination of failure data does not affect the quality and reliability of the final data processing results as data are obtained continuously and observations can be repeated with a frequency better than 6 days, considering two satellites (Sentinel 1A and 1B) and both ascending and descending passes [40].

### 3.2. Experimental Datasets

Due to these advantages of Sentinel-1 data products, in summary, high and free of charge availability, wide ground coverage and fine resolution, these data are used in developing the automatic monitoring system for high-energy para-seismic events and its influence on the surface in the study area, the Żelazny Most, with the use of GNSS, seismic and PSInSAR techniques. The Żelazny Most is the largest tailings storage facility in Europe and the second largest in the world [44,45]. It is an integral part of copper production technological chain [46] that since 1977 collects the tailings from the three mines of the KGHM Polska Miedź (formerly - the Polish State Mining and Metallurgical Combine) [45]. This project exploits Level-1 SLC products in IW mode. Images comprising the area of interest are regularly acquired since September 2018 and processed using PSInSAR technique.

RFI characteristic depends strongly on the geographic position of data acquisition [2]. According to the RFI power map with continental coverage over Europe [38] our study area was located in potential RFI-affected zone. On the other hand, the IEEE GRSS RFI Observations Display System [47] did not indicate any irregularities in C-band frequency (ranging from 4–8 GHz) over this area.

In order to carry out experiments and to verify the proposed approach for RFI artefacts detection, we used 3 different quick-look datasets, named RIYADH, MOSCOW and ASMOW, for the needs of our research. This selection was based on above mentioned RFI power map and IEEE GRSS RFI Observations Display System. Each collection includes both correct images and images with various levels of RFI contamination. RIYADH dataset consists of 136 images that were collected between May 2015 and January 2019, and comprise the area of the capital of Saudi Arabia. The MOSCOW set includes 99 images acquired between January and December 2019. These images cover the area surrounding the capital of Russia. The ASMOW collection consists of 53 images covering the study area, located in southwest Poland, in Lower Silesia, east of the town of Polkowice in the municipality of Rudna. These data were acquired from September 2018 to February 2020.

## 4. Methodology

In this section, we introduce basic information about the techniques we used in the experimental part. Here we start by discussing an example of how to store an image in digital form.

### 4.1. Digital Representation of the Image

A digital image can be simply represented by binary numbers of pixel color saturation in the relevant system [48,49]. Greyscale image pixels are represented by single Bytes that take decimal values from 0 to 255. In binary format from 00000000 to 11111111. Sample change 01101011⇒01101010 would not be noticeable to the human eye and in that way we could exploit that shortcoming of a human eye to hide data. RGB image pixels are represented by triples of Bytes which take decimal values from 0 to 255. For example, white color in decimal form is represented by (255,255,255) and in binary form by (11111111,11111111,11111111). Data in digital systems are often shown in hexadecimal form for reading convenience. The white color is (FF,FF,FF) where *F* is the letter of the hexadecimal system alphabet to which a decimal value of 15 is assigned.

In the pre-processing step, the images are converted to greyscale representation. This conversion makes it very easy to use feature extraction techniques. Next, we discuss a sample conversion.

### 4.2. Conversion from RGB to Greyscale

(1) $$new_{pixel}=R_{old_{pixel}}*0.292+G_{old_{pixel}}*0.594+B_{old_{pixel}}*0.114$$

**Figure 1:**
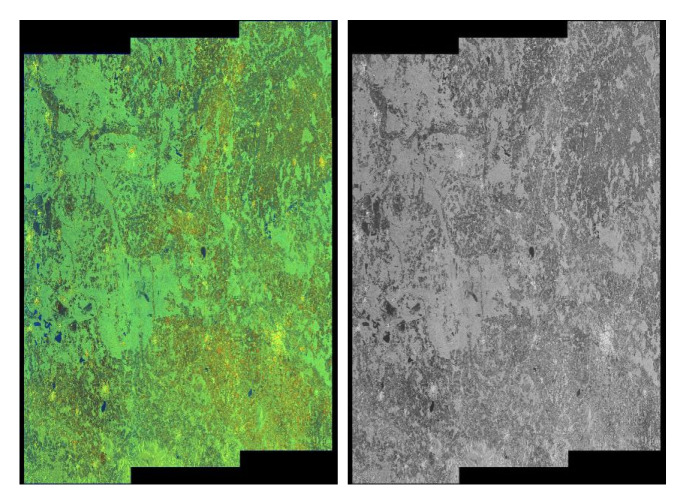
ASMOW quick-look—demonstration of conversion to greyscale using the formula (Section 4.2).

The above formula is a transformation from OpenCV library [50]. An example of a conversion is in Figure 1.

In the next section we discuss the technique of image features extraction based on pixel convolution [51,52,53].

### 4.3. Feature Extraction Based on Pixel Convolution

**Figure 2:**
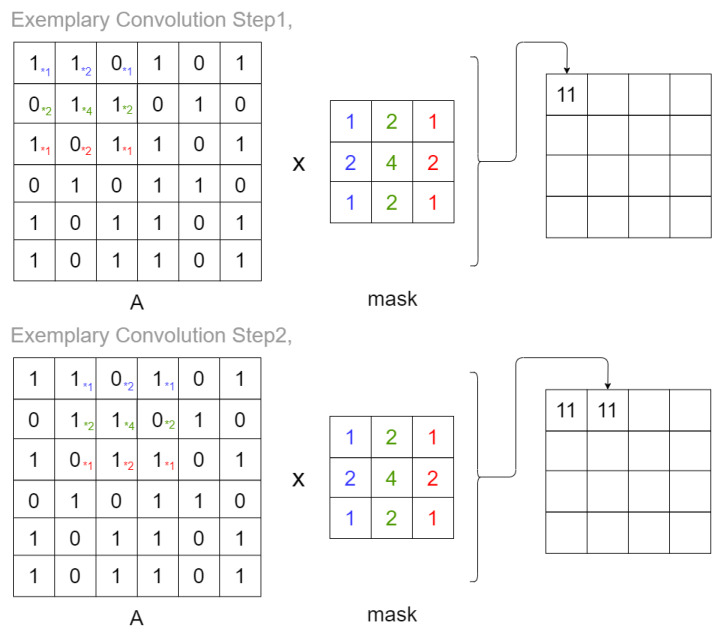
Exemplary convolution—first two steps based on Gaussian blur mask 3×3, $A_{x,y}\times mask=\sum_{i=1}^{mask\_width}\sum_{j=1}^{mask\_height}A_{x+i-1}*mask_{ij},$(x,y) are the top left coordinates of convolved pixels.

In Figure 2 we have an example of how a pixel convolution works. We have used the 3×3 Gaussian blur mask.

Other useful techniques that were applied are image filtering techniques by means of thresholding [51,52,54]. The method allowed us to focus on areas that are visible with a fixed, properly defined frequency or belonging to a defined range.

### 4.4. Thresholding

We used two types of thresholding [52]. The first type consists of giving the image pixels that do not exceed a certain fixed normalized threshold of color saturation.

The second method consisted of using a histogram of pixel values and filtering out those which (e.g., giving black color) belong to a fixed frequency range.

The last method we tested for an artefact classifier was the use of structural filtering with the nearest pixel neighbors [51,52,55,56].

### 4.5. Nearest Neighbor Structure Filtering

We also applied the technique of filtering the image structure by replacing the pixel with its closest neighbor in the sense of Manhattan distance [57,58]. Filtering allows you to eliminate unstructured pixels in the image. This step allowed us to better discriminate against images with artefacts.

As a reference classifier defined in the following section, we used the Convolutional Neural Network (CNN) [59,60].

### 4.6. Exemplary Deep Neural Network Architecture as Referenced Classifier

To verify the effectiveness of our artefacts extraction method, we used a simple deep neural network [59,61] defined in this section for classification. We used Python related tools such as PyTorch, TorchVision and NumPy. The visualization of results was performed using Seaborn and Matplotlib. A transformation was made when loading images, scaling everything to 400 × 600 pixels to ensure the same size of the input for the network. The data were divided into train and test sets in 80/20 ration in a random way. A simple network with two convolutional layers, linear transformations and pooling was proposed. The activation function was RELU (f(coloursaturation)=max(0,coloursaturation)), and loss function took the form of categorical Cross Entropy (thus it can be higher than one).

The Adam optimizer [62] was used. The training was done over 15 epochs. To split the data, the following function was used:

train_dataset,test_dataset=torch.utils.data.random_split(dataset,[train_size,test_size])

A detailed definition of the neural network is given in Listing 1.

Listing 1 **Neural network configuration.**

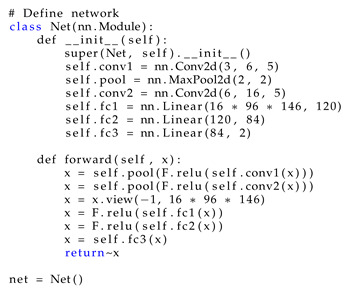

To make it easier to reproduce our research results, here are the versions of the libraries we installed to carry out the experiments (see Listing 2).

Listing 2 **Versions of the libraries used.**

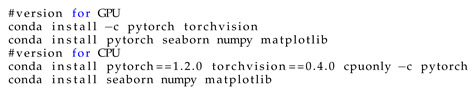

## 5. Experimental Session Details

To carry out the research we have used the bitmap library.hpp—see [63] library, which allows simple image processing. We used RIYADH (37 objects with strong defects, 72 without damages and 27 with weak artefacts), MOSCOW (53 correct, 26 difficult and 20 with strong artefacts) and ASMOW dataset (data from our project, we have only few defects; 50 correct objects and only 3 damaged) to check our methods. With RIYADH and MOSCOW collections, our aim was to find a pre-processing technique that will improve learning by means of a deep neural network. With ASMOW (due to its small class with artefacts), we were looking for a technique to effectively mark artefacts in an image, without classifying them with a network. In an experiment with deep neural network classification—see details in Section 4.6—the image sets were divided into a training subset where the network is taught and the validation test set, 20 percent of the objects on which the final neural network was tested. To estimate the quality of the classification on RIYADH and MOSCOW data we used the Monte Carlo Cross Validation [59,64] 5 technique (MCCV5, i.e., five times train and test), presenting average results. In the tests, we considered two binary classifications and one of three classes. In case of separation of three classes, in the network settings (from Section 4.6) we set the number of classes and we consider three outputs from the network.

### 5.1. Artefacts Detection in the RIYADH Dataset

**Figure 3:**
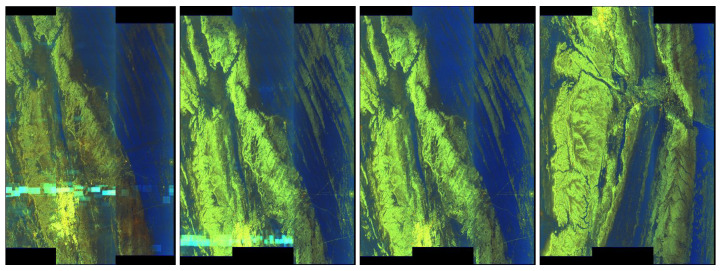
RIYADH quick-looks—exemplary pictures—the two on the left with artefacts, two on the right undamaged.

Consider the examples of problems with artefacts detection from our experimental groups of quick-look images. The first one is the RIYADH quick-look dataset. In Figure 3 we present sample images with artefacts and undamaged ones.

Next, we describe the steps of searching for our method of extracting artefacts on the RIYADH collection.

The first step was to convert the image into a greyscale form—according to the formula from Section 4.2.

In the next step, we tested the detectors of features based on pixel convolution.

### 5.2. Overview of Feature Detectors Based on Convolution

**Figure 4:**
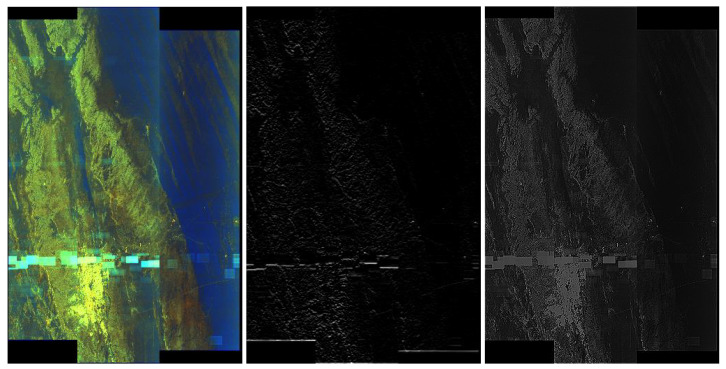
On the left side we have sample image for testing feature detectors. In the middle there is convolution based on Sobel’s gradient sharpening. On the right convolution based on Laplace filtering (sharpening).

**Figure 5:**
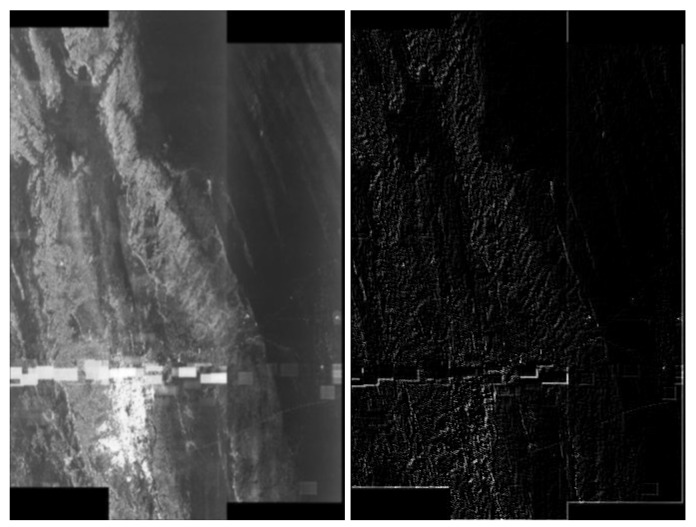
On the left side we have convolution based on Gaussian blur mask. On the right convolution based on Emboss mask.

In this section, we present the selected feature detectors on the basis of an image with artefacts—see the left picture (Figure 4). We treated this step as a pre-processing stage allowing us to extract specific features of images. For example, masks allowed us to create frames of artefacts potentially useful for shape detection in the image. Some examples of well-functioning masks in this context are seen in Figure 4 and Figure 5. Consider the effect of selected popular filters [49] usage: in the middle picture (Figure 4) we have Sobel’s gradient sharpening, in the right picture (Figure 4) we have Laplace filtering (sharpening), in the left picture (Figure 5) we have Gaussian blur mask and finally in the right picture (Figure 5) we have Emboss mask usage. According to our tests, Gaussian blur filter is one the best at extracting artefacts from the RIYADH images. We applied this mask to the hybrid technique together with thresholding.

**Figure 6:**
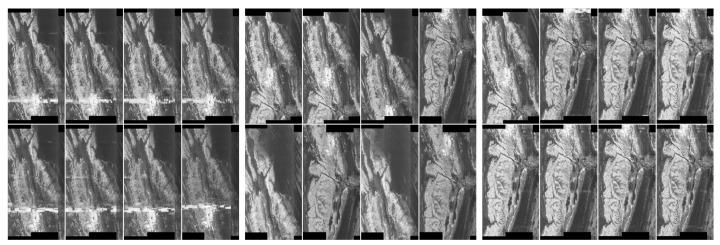
Comparison of the effects of Gaussian blur 3×3 convolution in the pictures, from the left: with artefacts, without artefacts and difficult—the discrimination of classes is not explicit.

We have considered the following feature detectors:
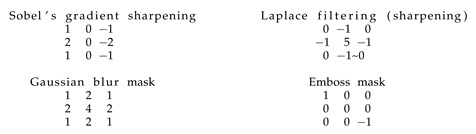


We provide more extensive testing of the Gaussian blur on the RIYADH data in Figure 6.

### 5.3. Application of Thresholding

**Figure 7:**
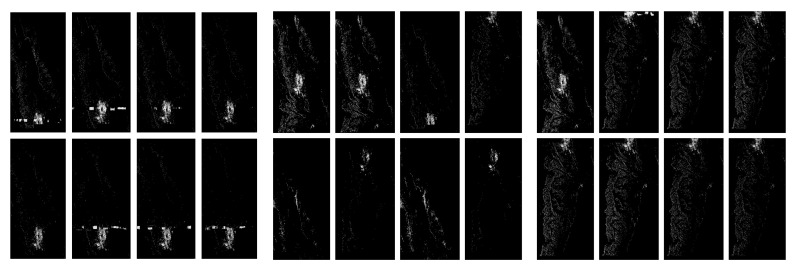
Comparison of the effects of thresholding in the pictures, from the left: with artefacts, without artefacts and difficult—the discrimination of classes is not explicit.

**Figure 8:**
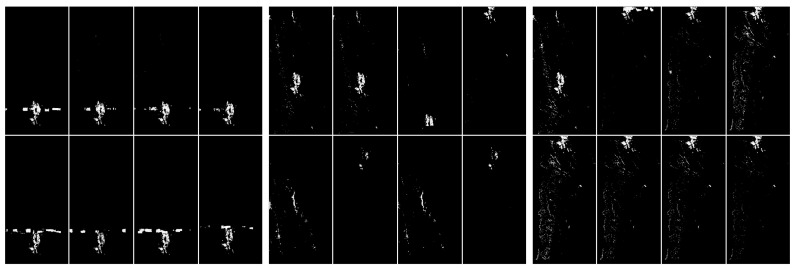
Demonstration of the application of two steps, the convolution with Gaussian blur 3×3 and thresholding 0.1, from the left: with artefacts, without artefacts and difficult. Example of a Python (Jupiter Notebook) thresholding. The level of artefacts separation is higher after these two steps. Classification may consist in calculating the narrowest possible uniform white surface.

**Figure 9:**
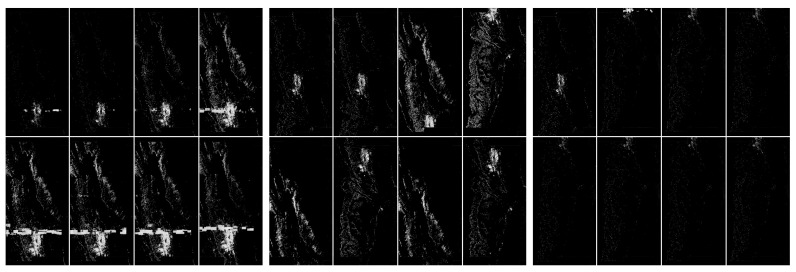
Visualization of the application of pixel frequency threshold. In this case a histogram is created for [0,255], and the frequency threshold is set to 300. All color values that occur more often are replaced by black. After thresholding on the basis of frequency, it is clear that the artefacts have become exposed; however, there are also many unnecessary white pixels in the picture. From the left: with artefacts, without artefacts and difficult.

Next, we discuss the results of experiments with the application of thresholding techniques from Section 4.4. In Figure 7 we have a demonstration of the first threshold method from Section 4.4. As we can observe, discrimination is not very accurate when using this technique alone. Using the hybrid method, combining Gaussian blur and thresholding—see Figure 8, we get a slightly better artefacts separation effect. The application of the second thresholding method based on the pixel histogram can be seen in Figure 9.

### 5.4. Application of Nearest Neighbor Filtering

**Figure 10:**
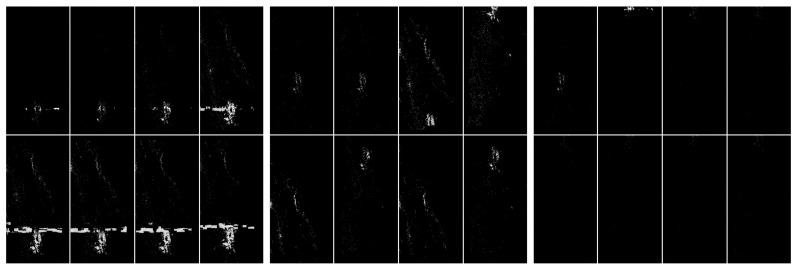
Visualization of the application of pixel frequency threshold and one nearest neighbor filtering. In this case, a histogram was created for [0,255], and the frequency threshold was set to 300. All color values that occur more often are replaced by black. The next step of the image processing was to swap pixels with their nearest neighbors. This step filtered out some unstructured pixels. The level of discrimination has clearly increased. From the left: with artefacts, without artefacts and difficult.

**Figure 11:**
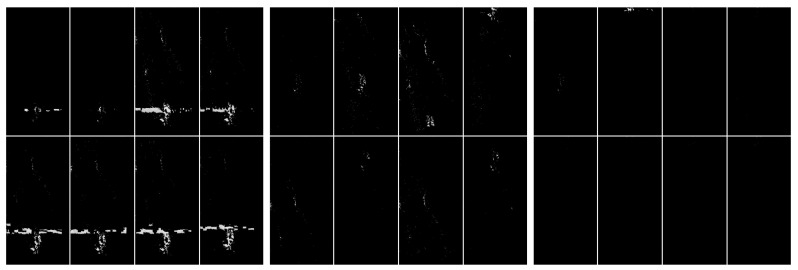
Visualization of the application of pixel frequency threshold and one nearest neighbor filtering. In this case a histogram is created for [0,255], and the frequency threshold is set to 300. All color values that occur more often are replaced by black. The next step of the image processing was to swap pixels with their nearest neighbors. In the present case, we have done the swap procedure twice. This step filtered out some unstructured pixels. The level of discrimination has clearly increased. From the left: with artefacts, without artefacts and difficult.

In Figure 10 and Figure 11, we present hybrid methods which involved thresholding based on pixel histogram and noise filtering technique using the nearest neighbors of pixels. The use of these methods clearly shows the possibility of separating artefacts.

### 5.5. Summary of Results for Detection Artefacts in RIYADH Dataset

**Figure 12:**
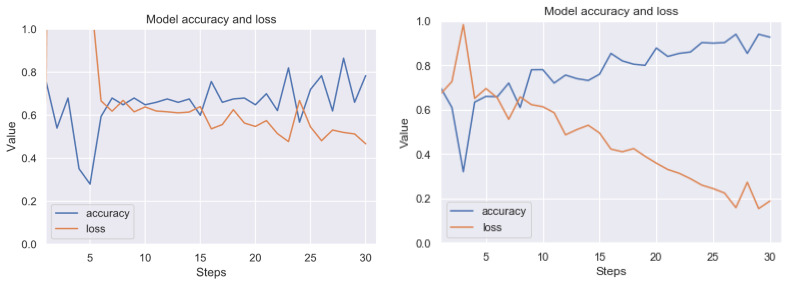
Exemplary learning effect before (left side) and after using our technique (right side). Result for RIYADH dataset. Class ok (without artefacts) vs. class er (with strong artefacts). The line graphs shows accuracy (blue) and loss (orange) for 15 epochs (30 steps from batch). Although the image dataset is relatively small for Convolutional Neural Network (CNN), there can be observed a good trend in increasing accuracy, while minimizing loss.

**Figure 13:**
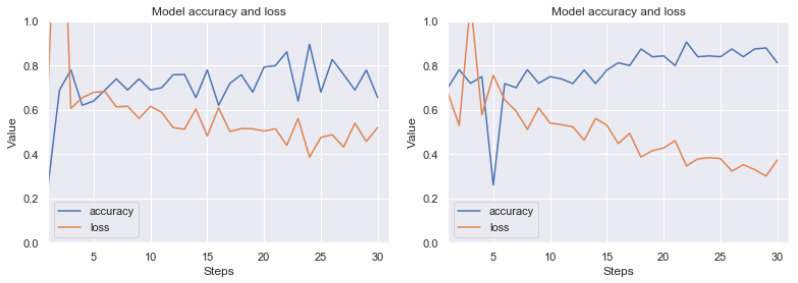
Exemplary learning effect before (left side) and after using our technique (right side). Result for RIYADH dataset. Class ok (without artefacts) vs. class difficult (with weak artefacts). The line graphs shows accuracy (blue) and loss (orange) for 15 epochs (30 steps from batch). Although the image dataset is relatively small for CNN, there can be observed a good trend in increasing accuracy, while minimizing loss.

**Figure 14:**
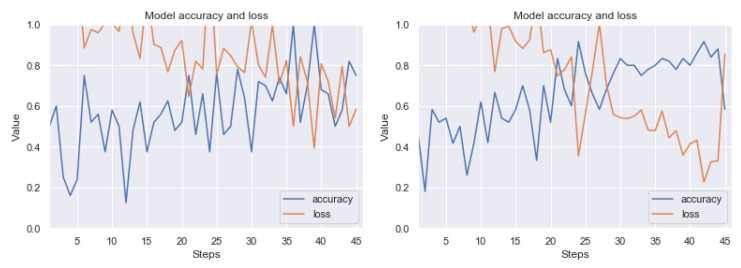
Exemplary learning effect before (left side) and after using our technique (right side). Result for RIYADH dataset. Class ok (without artefacts) vs. class er (with strong artefacts) vs. class difficult (with weak artefacts). The line graphs shows accuracy (blue) and loss (orange) for 15 epochs (30 steps from batch). Although the image dataset is relatively small for CNN, there can be observed a good trend in increasing accuracy, while minimizing loss. The accuracy of classification on the validation set came out nearly 55 percent.

Summarizing the results obtained, we can state that the most effective technique of detecting artefacts, from among the ones we have studied, is the use of thresholding based on histograms of pixel values and noise filtering using the closest neighbors method. The result of good performance of this combination was predictable, because artefact colors usually have a low frequency in pixel histograms; therefore, it is quite easy to visualize clear artefacts. Then the filtering method allows us to remove single, unstructured pixels. Let us present the results (before and after the application of our method) on the RIYADH dataset using the neural network described in Section 4.6. The exemplary epochs of learning for class ok (without artefacts) and er (with strong artefacts) (before and after the application of our method) is available in Figure 12. A similar result for classification of ok class and class difficult (with weak artefacts) is in Figure 13. In Figure 14 we have learning between the three mentioned classes. The exact results from the MCCV5 test can be seen in Table 1. The results are promising, considering the separation of the undamaged image class from the heavily damaged ones—the reference classification level was about 74 percent, and after applying our method, the degree of class distinction (artefacts detection) increased to a level close to 92 percent of accuracy. Similarly, the detection accuracy of weak artefacts has increased from about 68 percent to 84 percent. A spectacular increase can be observed in the level of discrimination between undamaged images and weak and strong artefacts from 54 percent to nearly 81 percent on validation set. Seeing Figure 12, Figure 13 and Figure 14, the process of learning the neural network after applying our method seems to be more stable. Standard deviation (see Table 1) without pre-processing reaches 13 percentage points, after applying our method, it is within 5, 6 percentage points for variants (er vs. ok), and classifying all three classes. In case of the classification of weak artefacts is it similar in both cases within 8 percentage points.

Next, we discuss the results on artefacts collection from the MOSCOW database.

### 5.6. Results for MOSCOW Dataset

**Figure 15:**
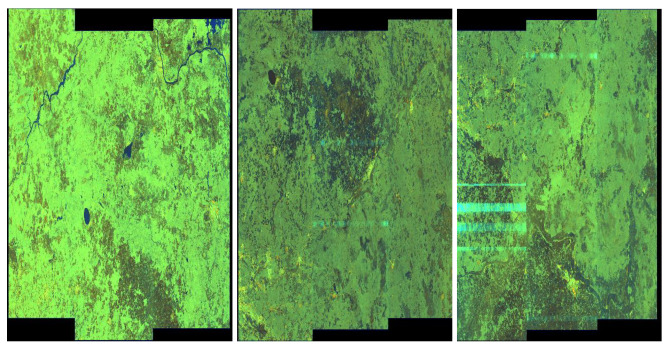
MOSCOW quick-looks—exemplary pictures, from the left to right, without artefacts (class *0*), with weak artefacts (class *I*) and with strong artefacts (class II).

We considered three classes: *0* (no artefacts), *I* (weak artefacts) and *II* (strong artefacts)—see Figure 15. We conducted experiments with similar network settings as in Section 5.1. The size of classes *0*, I and II was 53,26 and 20, respectively.

**Figure 16:**
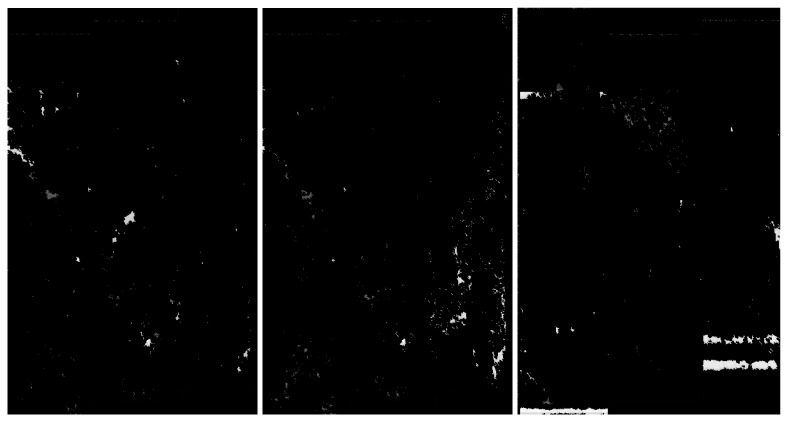
MOSCOW quick-looks—exemplary pictures, from the left to right, without artefacts (class *0*), with weak artefacts (class *I*) and with strong artefacts (class II).

We applied the same steps as for the RIYADH dataset. We used a frequency range of 1000 for thresholding. Samples of data after applying our method on the MOSCOW collection can be seen in Figure 16.

Next, we briefly present our results.

### 5.7. Summary of Results for MOSCOW Dataset

**Figure 17:**
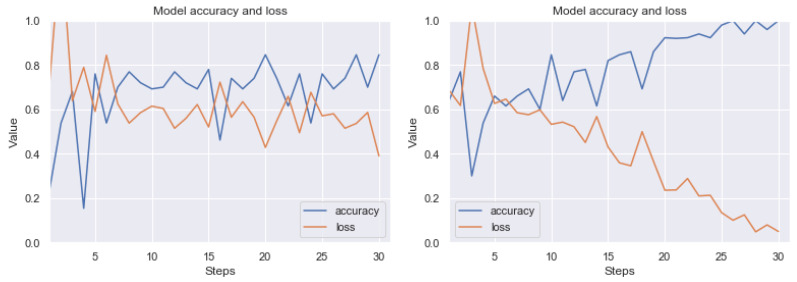
Exemplary learning effect before (left side) and after using our technique (right side). Result for MOSCOW dataset. Class *0* (without artefacts) vs. class *I* (with weak artefacts). The line graphs shows accuracy (blue) and loss (orange) for 15 epochs (30 steps from batch). Although the image dataset is relatively small for CNN, there can be observed a good trend in increasing accuracy, while minimizing loss.

**Figure 18:**
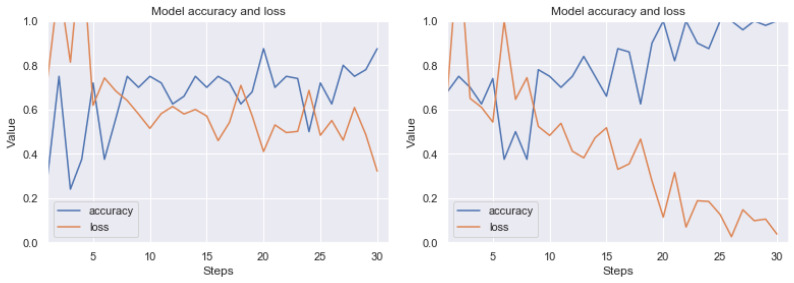
Exemplary learning effect before (left side) and after using our technique (right side). Result for MOSCOW dataset. Class *0* (without artefacts) vs. class II (with strong artefacts). The line graphs shows accuracy (blue) and loss (orange) for 15 epochs (30 steps from batch). Although the image dataset is relatively small for CNN, there can be observed a good trend in increasing accuracy, while minimizing loss.

**Figure 19:**
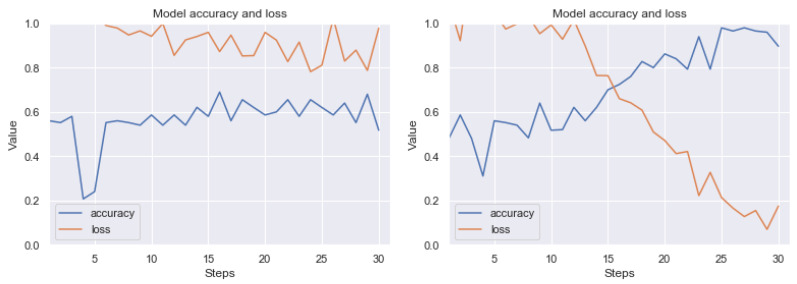
Exemplary learning effect before (left side) and after using our technique (right side). Result for MOSCOW dataset. Class *0* (without artefacts) vs. class *I* (with weak artefacts) vs. class *II* (with strong artefacts). The line graphs shows accuracy (blue) and loss (orange) for 15 epochs (30 steps from batch). Although the image dataset is relatively small for CNN, there can be observed a good trend in increasing accuracy, while minimizing loss.

Detailed test results using the MCCV5 method are shown in Table 2. An example of the learning effect on these data before and after the application of our method can be seen in Figure 17, Figure 18 and Figure 19. The results are not as good as those of RIYADH, but they indicate the positive effects of our method. For large artefacts, efficiency has increased from 61 percent to 80 percent after using our method. The results for the classification of weak artefacts (class *I*) are comparable on the validation set. Despite the fact that the quality of neural network learning on three classes has increased significantly (see Figure 19), the classification on the validation set increased by around 8 percentage points. Standard deviation (see Table 2) without pre-processing reaches around 15 percentage points, after applying our method, it is within 5, 6 percentage points for variants (II vs. *0*) and classifying all three classes. In case of the classification of weak artefacts the results are comparatively low with high standard deviation up to 18 percentage points.

Let us move on to test the ASMOW collection, an important one for our project.

### 5.8. Results for ASMOW Dataset

In this section, we describe the detection of artefacts, which appear in the ASMOW quick-look dataset. Performing referenced deep learning classification was not possible due to the availability of RFI-affected images. From here we simply present the procedure for detecting artefacts on this data—with the position of the artefact marked in the picture.

**Figure 20:**
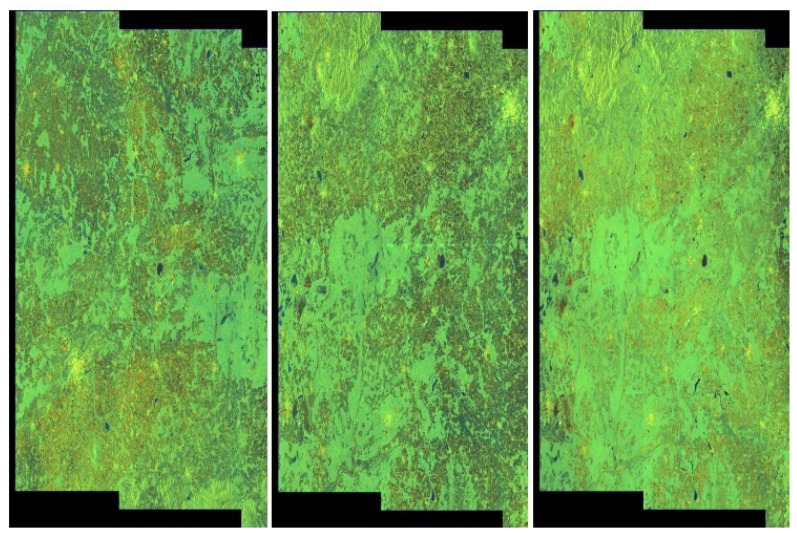
Example of original data from the ASMOW quick-look dataset, there is a sample with an artefact in the middle.

**Figure 21:**
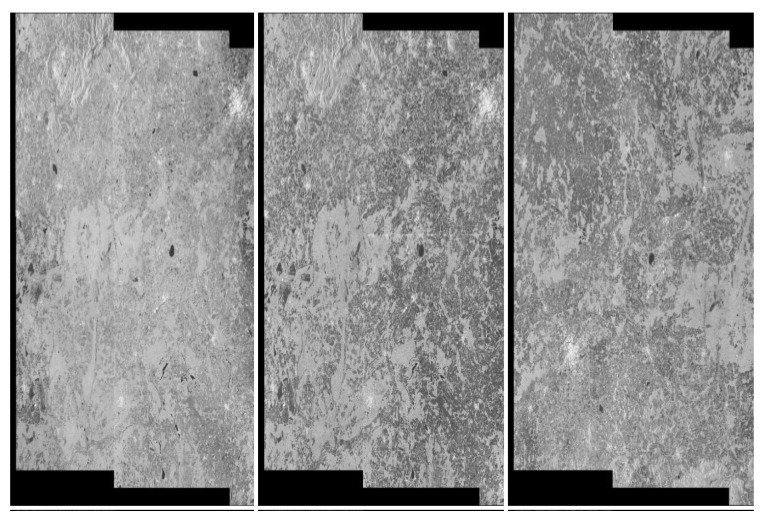
Step1: We carried out the pixel convolution with the Mask14 (Gaussian blur), on the data from Figure 20.

**Figure 22:**
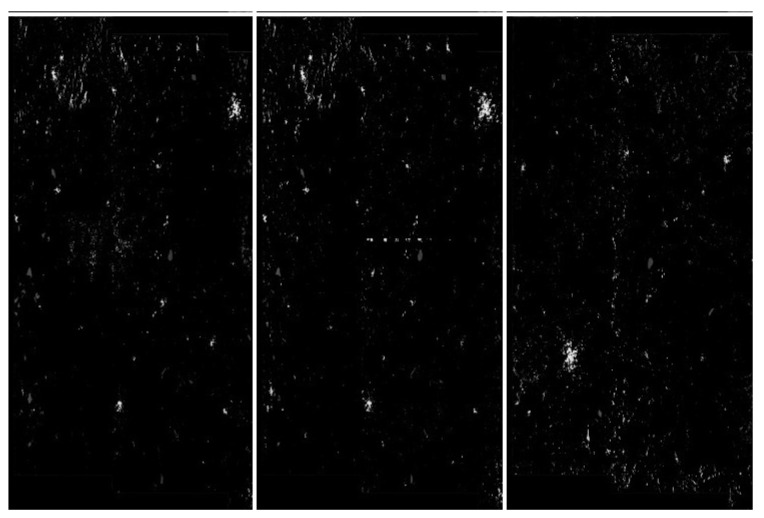
Step2: The thresholding described in Section 4.4 and the noise reduction by method 1 nn described in Section 4.5.

**Figure 23:**
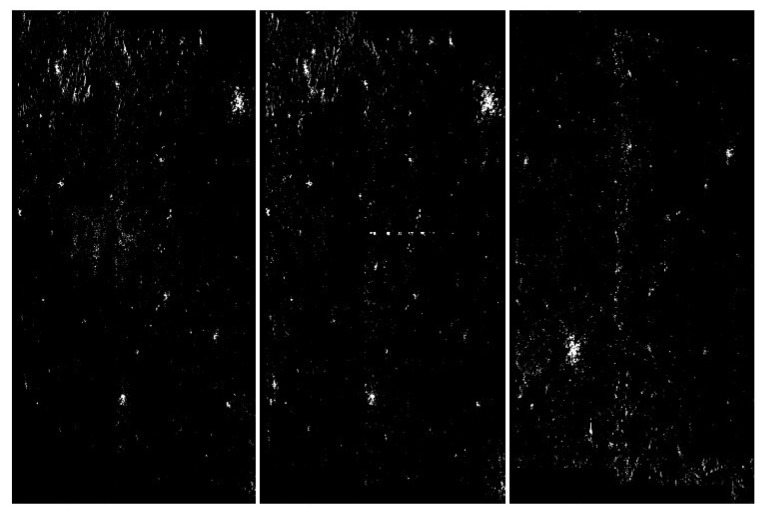
Step3: In this step, we binarized the pixels for better recognition of the artefact.

**Figure 24:**
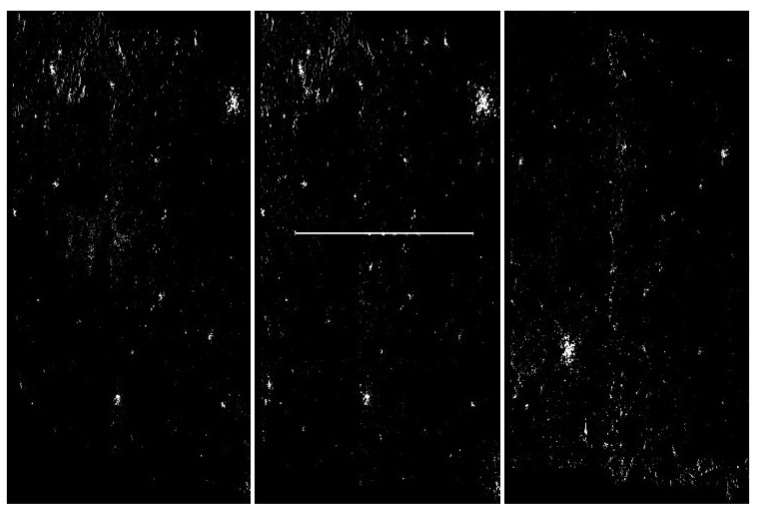
Step4: In the last step, we detected the artefact: a horizontal plane that passes unevenly through the line. We established that we are looking for a sufficiently long plane, consisting of seven-pixel blocks passing horizontally through the image at the same height. Our acceptance threshold for detecting the artefact, resulting from the experiments, is 40. So the artefact consists of a minimum of 40 seven-pixel blocks arranged horizontally.

The detection procedure is as follows:

1. Considering sample data where the middle picture contains an artefact—see Figure 20. We convolved the pixels using the Mask14 (Gaussian blur) see Figure 21.
2. We applied a hybrid threshold method based on the histogram (with a fixed frequency threshold of 10,000), and using the 1 nn technique for noise reduction, see Figure 22.
3. We have applied pixel binarization [65], setting the threshold of 120, every pixel with saturation less than 120 is black, the rest are white, see Figure 23.
4. Then we detected the area whose pixels are arranged in a thick straight line—see Figure 24. The thickness was set at 7 vertical pixels. We applied the expected linear structure length threshold of 40, so our artefact was detected and distinguished from undamaged images. The area of artefact was automatically marked with a line.

### 5.9. Summary of Results for ASMOW Dataset

The experimental results show that the detection of these very little visible artefacts requires the following steps. The first two are analogous to the detection of the artefacts of RIYADH dataset, that is, (1) we use pixel convolutions by means of Gaussian blur, (2) thresholding on the basis of the color occurrence frequency, (3) defrosting using the 1 nn technique, (4) binarization and (5) detection of a plane consisting of pixel blocks. Artefacts can be successfully detected as we can see in Figure 24.

## 6. Conclusions

In this work, we tested a group of image processing techniques to separate clear RFI artefacts from undamaged images. We reviewed the masks used to select features in the process of convolution. Then we tested methods of thresholding. The first one is based on the selection of pixels in a fixed standardized range. The second one involves filtering pixels that do not meet the established criterion of color frequency (based on color histogram). Then we tested the hybrid solution with thresholding and filtration method based on the nearest neighbor of pixels. We verified the results of our methods of separating artefacts using a convolutional neural network as a reference classifier. The classification was carried out on raw data and on data prepared by our methods—with the Monte Carlo Cross Validation 5 model. In our work, we considered three datasets with artefacts. The first one comes from RIYADH, the second one from MOSCOW and the third one from ASMOW quick-look dataset. In case of RIYADH and MOSCOW datasets we see that for the separation of our RFI artefacts, the best level of their separation is given by the Gaussian blur. The best method, among the tested ones, that gives a clear separation of large artefacts is a hybrid of frequency based on thresholding and filtration with the nearest neighbors method. In case of the ASMOW dataset of RFI artefacts, we applied the same steps as with RIYADH and MOSCOW, additionally applying binarization and detection of the plane arranged horizontally on the image consisting of pixel blocks. The initial goal was achieved, we found an easy to implement method of separating large RFI artefacts—not repairable with image filtering methods. The proposed solution can effectively support the automatic PSInSAR processing workflow by recognizing RFI-affected data, and as a consequence, removing them from the stack of SAR images required to determine the ground displacements. Our method improves (compared to the classification on raw data) the efficiency of artefact detection by up to 27 percentage points depending on the classification context under consideration. The standard deviation of the results after application of our methods is nearly 5,6 percentage points (except for the unstable classification of weak artefacts). The obvious conclusion: it is difficult to find a method to generalize the problem of searching for artefacts. Each dataset containing artefacts should be treated individually. The model structure should be selected in a personalized way.

One of the methods of developing our detection system was the use of techniques for the recognition of specific shapes relevant to the appearing RFI artefacts and the use of complex convolutional neural networks—which is the foreground of our future research.

## Figures and Tables

**Table 1:** Summary of results for RIYADH–MCCV5 technique; nil.ok.er = accuracy of classification of er and ok classes before pre-processing, ok.er = accuracy of classification of er and ok classes after application of our method, nil.ok.tr, ok.tr, nil.all, all = analogous parameters, showing the class separation before and after application of our technique, SD = standard deviation of results, avg = average result.

Test No.	nil.ok.er	ok.er	nil.ok.tr	ok.tr	nil.all	All
1	0.8181	1.0	0.7	0.8571	0.5358	0.7586
2	0.7727	0.8696	0.7	0.9048	0.3928	0.8621
3	0.5	0.9130	0.75	0.7143	0.5714	0.8621
4	0.7727	0.9565	0.55	0.8571	0.6071	0.7586
5	0.8181	0.8696	0.7	0.9048	0.6071	0.8276
**avg**	0.73632	0.92174	0.68	0.84762	0.54284	0.8138
**SD**	0.134042986	0.056671051	0.075828754	0.078251307	0.088932463	0.052321841

**Table 2:** Summary of results for MOSCOW–MCCV5 technique; nil.ok.er = accuracy of classification of er and ok classes before pre-processing, ok.er = accuracy of classification of er and ok classes after application of our method, nil.ok.tr, ok.tr, nil.all, all = analogous parameters, showing the class separation before and after application of our technique, SD = standard deviation of results, avg = average result.

Test No.	nil.0.II	0.II	nil.0.I	0.I	nil.all	All
1	0.533	0.8	0.563	0.5	0.55	0.7
2	0.467	0.8	0.563	0.5	0.6	0.6
3	0.733	0.866	0.5	0.937	0.45	0.55
4	0.8	0.8	0.563	0.63	0.5	0.6
5	0.533	0.733	0.813	0.75	0.55	0.6
**avg**	0.613	0.8	0.6	0.663	0.53	0.61
**SD**	0.145	0.047	0.122	0.185	0.057	0.055
